# Potential association between chronic alpha-1 adrenergic blockade and early epinephrine-related resuscitation profiles during cardiac arrest: a retrospective exploratory cohort study

**DOI:** 10.3389/fcvm.2026.1888582

**Published:** 2026-08-07

**Authors:** Reşad Beyoğlu, Filiz Özyiğit

**Affiliations:** Department of Emergency Medicine, Bandirma Onyedi Eylül University, Bandirma, Türkiye

**Keywords:** alpha-1 adrenergic blockade, cardiac arrest, epinephrine, resuscitation pharmacology, translational pharmacology, vasopressor responsiveness

## Abstract

**Background and objectives:**

Epinephrine remains central to advanced cardiac life support through combined alpha- and beta-adrenergic effects. Alpha-mediated vasoconstriction increases aortic diastolic and coronary perfusion pressures, while beta-adrenergic, particularly beta-2, actions may contribute to pulse generation and ROSC. This study explored whether chronic alpha-1 blocker therapy is associated with early resuscitation profiles in epinephrine-treated cardiac arrest patients.

**Materials and methods:**

This retrospective cohort study included adults who received intravenous epinephrine during cardiac arrest between 2014 and 2025. The primary exploratory endpoint was operationally defined refractory arrest, based on cumulative epinephrine dose ≥3 mg and/or CPR duration ≥20 min without sustained ROSC, and was assessed separately from failure to achieve sustained ROSC. Baseline imbalance was evaluated using standardized mean differences. Because the exposed group was small, a prespecified parsimonious logistic regression model adjusted for age, sex, arrest location, and initial rhythm.

**Results:**

Among 2,472 patients, 74 (3.0%) were receiving chronic alpha-1 blocker therapy; all included patients received epinephrine during CPR. Refractory arrest, distinct from ROSC failure, occurred in 45/74 (60.8%) alpha-blocker users versus 840/2,398 (35.0%) non-users (adjusted OR 3.06, 95% CI 1.88–4.97). Failure to achieve sustained ROSC occurred in 47/74 (63.5%) versus 1,002/2,398 (41.8%) patients (adjusted OR 2.64, 95% CI 1.62–4.31), while ROSC was lower among alpha-blocker users (adjusted OR 0.38, 95% CI 0.23–0.62). Early mortality occurred in 54/74 (73.0%) versus 636/2,398 (26.5%) patients (adjusted OR 7.15, 95% CI 4.20–12.15). Meaningful baseline imbalance was present, particularly for coronary artery disease and age (SMD 1.619 and 1.087, respectively).

**Conclusions:**

Chronic alpha-1 blocker therapy was associated with unfavorable early resuscitation outcomes among epinephrine-treated cardiac arrest patients. These exploratory findings do not establish causality and should be interpreted cautiously because of baseline imbalance, possible residual confounding, incomplete resuscitation-level covariates, and the small exposed group. Further multicenter studies with richer physiologic, pharmacologic, and statistical characterization are needed.

## 1 Introduction

Cardiac arrest remains one of the most physiologically extreme conditions encountered in acute care medicine and continues to be associated with substantial morbidity and mortality despite ongoing advances in resuscitation science. During cardiopulmonary resuscitation (CPR), restoration of coronary and cerebral perfusion is essential for return of spontaneous circulation (ROSC) and early survival. Epinephrine remains a cornerstone of advanced cardiac life support, and its alpha-1 adrenergic receptor-mediated vasoconstrictive effects increase aortic diastolic pressure and coronary perfusion pressure during CPR (1, 2).

The hemodynamic and electrophysiological effects of epinephrine during cardiac arrest arise from combined alpha- and beta-adrenergic receptor activation rather than from alpha-1 stimulation alone. Alpha-adrenergic vasoconstriction increases aortic diastolic pressure and coronary perfusion pressure, which are widely regarded as key determinants of ROSC (1, 2). At the same time, beta-adrenergic stimulation, and beta-2 adrenoceptor activation in particular, appears to contribute meaningfully to pulse generation in the sinoatrial node and to restoration of contractile function in ischemic myocardium, a mechanism that has been proposed as part of the rationale for epinephrine's continued preference over pure alpha-1 agonists such as phenylephrine during CPR (3). Beta-adrenergic stimulation has also been linked to potentially unfavorable effects, including increased myocardial oxygen consumption, post-resuscitation myocardial dysfunction, and arrhythmogenesis (4–6). The clinical significance of epinephrine during cardiac arrest therefore reflects a balance between beneficial alpha-1-mediated vasoconstriction and dual beta-adrenergic effects that may both support pulse generation and predispose to post-resuscitation complications; this balance, rather than alpha-1 activity in isolation, forms the physiological backdrop against which the present findings should be interpreted (3–6).

Alpha-1 adrenergic blockers, including doxazosin, alfuzosin, and silodosin, are widely prescribed for benign prostatic hyperplasia and hypertension, particularly in older adults. By competitively antagonizing peripheral alpha-1 receptors, these agents reduce vascular tone and may theoretically alter responsiveness to endogenous and exogenous catecholamines. Although this interaction has been investigated in several physiological and hemodynamic settings, its potential relevance during cardiac arrest remains insufficiently characterized (7, 8). From a mechanistic perspective, chronic alpha-1 blockade could plausibly influence the alpha-mediated component of epinephrine's vasopressor effect during CPR, especially under conditions of impaired tissue perfusion and reduced drug delivery inherent to cardiac arrest physiology, while the beta-adrenergic component of epinephrine's action would be expected to remain unaffected by alpha-1 blockade (8).

In recent years, increasing attention has been directed toward refractory or vasopressor-resistant cardiac arrest states, particularly in the context of prolonged resuscitation and extracorporeal cardiopulmonary resuscitation strategies (9–11). However, most previous investigations have focused on resuscitation timing, mechanical support strategies, or post-arrest care, while relatively little attention has been given to chronic outpatient pharmacologic factors that may influence vasopressor responsiveness during CPR. Although several observational studies have examined the relationship between pre-arrest beta-blocker therapy and cardiac arrest outcomes, the findings remain inconsistent, and the potential impact of chronic alpha-1 adrenergic blockade has been largely overlooked (9–13).

Importantly, patients receiving alpha-1 adrenergic blockers do not necessarily represent the highest-risk cardiovascular subgroup *a priori*, as these medications are commonly prescribed for benign prostatic hyperplasia and chronic hypertension. In addition, in some patients, alpha-1 adrenergic blockers may have been prescribed for non-cardiovascular indications or ophthalmology-related clinical contexts, further supporting the heterogeneity of the exposed group. This raises the possibility that any observed difference in resuscitation profiles may reflect not only differences in baseline patient characteristics, but also a potentially relevant pharmacodynamic interaction; as detailed in the Methods and Results, the present cohort in fact demonstrated marked baseline imbalance between exposure groups, which is addressed explicitly through standardized mean differences and adjusted modeling.

In this context, the present study aimed to explore whether chronic alpha-1 adrenergic blocker therapy was associated with altered early resuscitation profiles among adult cardiac arrest patients treated with epinephrine. Rather than attempting to establish causality, the study was designed to investigate a potentially relevant and underexplored pharmacodynamic signal within a large real-world resuscitation cohort, while explicitly acknowledging that beta-adrenergic mechanisms of epinephrine action, resuscitation-level covariates, and residual confounding may all contribute to the observed associations.

## 2 Materials and methods

### 2.1 Study design and setting

This study was designed as a single-center, retrospective cohort analysis conducted within the emergency department and the affiliated prehospital emergency medical services network of Bandırma Onyedi Eylül University Faculty of Medicine, Balıkesir, Türkiye. The study site is a high-volume emergency department with approximately 300,000 annual patient visits. All adult cardiac arrest cases managed between October 1, 2014, and October 1, 2025, were reviewed using electronic medical records and standardized resuscitation documentation. The study was conducted in accordance with institutional ethical standards.

### 2.2 Study population

All consecutive adult patients (≥18 years) who experienced out-of-hospital cardiac arrest (OHCA) or in-hospital cardiac arrest (IHCA) and received at least one dose of intravenous epinephrine during cardiopulmonary resuscitation (CPR) were screened for eligibility. Because receipt of at least one dose of intravenous epinephrine during CPR was itself a mandatory inclusion criterion, all 74 chronic alpha-1 blocker users and all 2,398 non-users in the final analytic cohort received intravenous epinephrine during resuscitation; no patient was analyzed without confirmed epinephrine exposure.

### 2.3 Inclusion criteria

- Age ≥18 years
- Documented cardiac arrest (OHCA or IHCA)
- Administration of at least one dose of intravenous epinephrine during CPR
- Available medication history allowing determination of alpha-1 adrenergic blocker use
- Sufficient documentation of arrest characteristics and outcomes

### 2.4 Exclusion criteria and flow of participants

Patients were excluded if any of the following were present:

- Missing key clinical or outcome data
- Incomplete medication records precluding exposure classification
- Traumatic cardiac arrest
- Known do-not-resuscitate (DNR) order prior to the arrest

**Figure 1:**
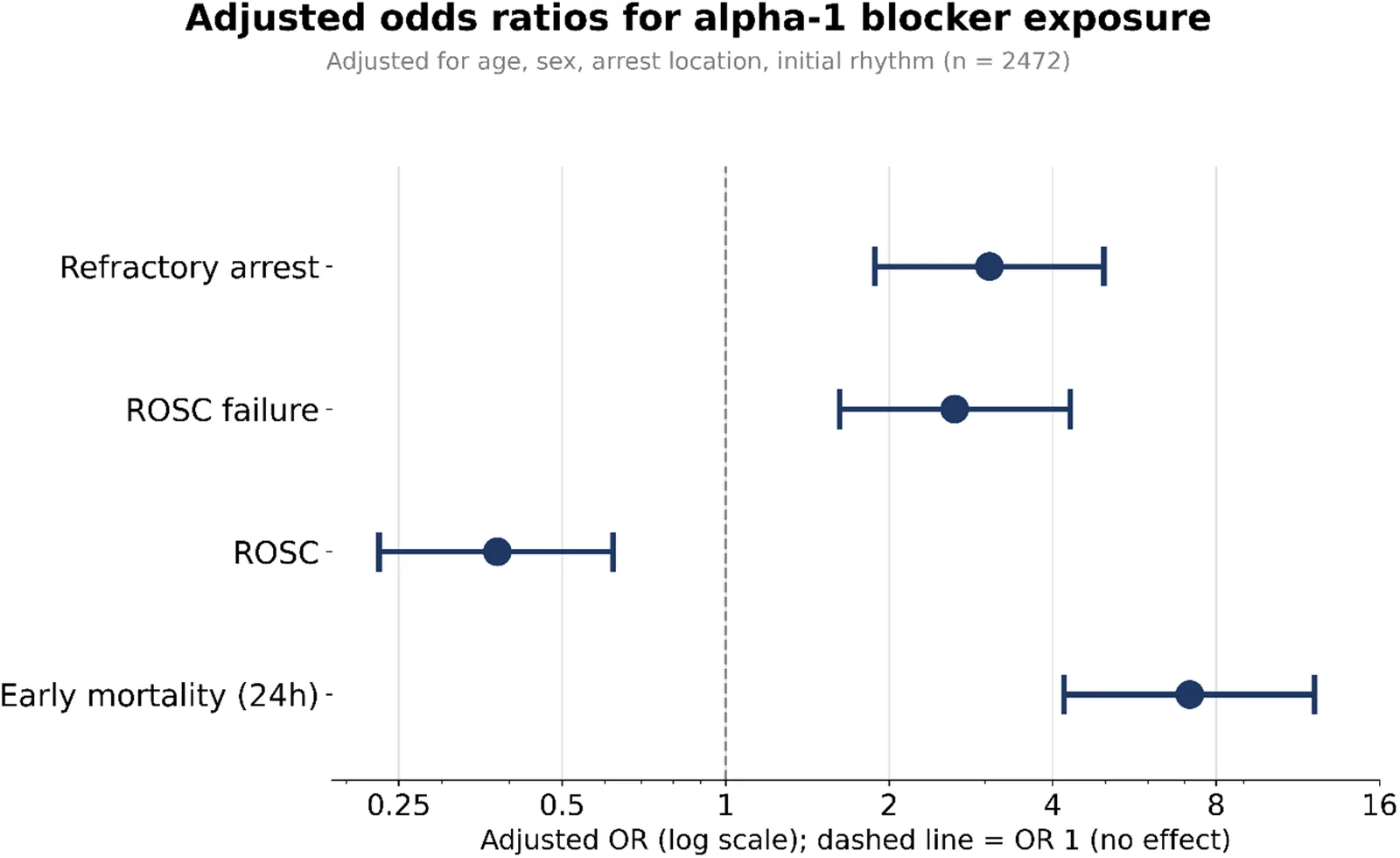
Flow diagram of patient screening, exclusion, and final inclusion, with exclusion reasons disaggregated into seven specific categories as requested.

Of 3,168 cardiac arrest cases screened, 696 were excluded, resulting in a final analytic cohort of 2,472 patients. In response to reviewer requests for a more granular accounting of exclusions, the single “incomplete clinical or outcome data” category has been disaggregated into specific reasons: no intravenous epinephrine administered during CPR (*n* = 173), missing outcome data (*n* = 146), missing arrest characteristics (*n* = 114), medication history unavailable or exposure could not be classified (*n* = 96), traumatic cardiac arrest (*n* = 70), known DNR order before arrest (*n* = 66), and duplicate, transfer, or otherwise unclear records (*n* = 31). The revised flow diagram (Figure 1) presents this disaggregated breakdown.

### 2.5 Exposure definition

The primary exposure of interest was chronic outpatient use of an oral alpha-1 adrenergic blocker at the time of cardiac arrest. For the purposes of this study, chronic use was defined as documentation of an active home medication or outpatient prescription before the arrest event. Patients with only newly initiated in-hospital therapy or unclear duration of use were not classified as exposed. Medication status was determined from all available sources, including prior hospital records, outpatient prescription data accessible through the electronic record, emergency referral documents, and medication reconciliation notes obtained from relatives or caregivers when available. Cases in which medication history could not be verified sufficiently were excluded from the analytic cohort. Alpha-1 adrenergic blockers included doxazosin, alfuzosin, and silodosin. Patients receiving any of these agents were categorized as alpha-blocker users; all remaining patients constituted the comparison group.

In routine clinical practice, alpha-1 adrenergic blockers are prescribed for long-term use; therefore, documentation of an active outpatient prescription was considered a reasonable proxy for chronic exposure. However, this operational proxy does not confirm active pharmacological exposure at the moment of arrest: neither medication adherence nor the exact timing of the last dose relative to the arrest event could be verified. Because doxazosin, alfuzosin, and silodosin differ in pharmacokinetic profile and alpha-1 receptor subtype selectivity, a descriptive agent-level breakdown is reported in Table 1; however, the small number of patients receiving each individual agent (27 doxazosin, 21 alfuzosin, 19 silodosin, 7 unknown/unspecified agent) precluded a statistically reliable agent-specific subgroup analysis, and this limitation is acknowledged explicitly in the Discussion.

**Table 1:** Missingness of key resuscitation and exposure-classification variables in the screened and final analytic cohorts.

Variable	Missing in screened cohort (*n* = 3,168)	Missing in final cohort (*n* = 2,472)	Missing in alpha-blocker (+) (*n* = 74)	Missing in alpha-blocker (–) (*n* = 2,398)	*P* value for missingness difference
CPR duration	407 (12.8)	0 (0.0)	0 (0.0)	0 (0.0)	N/A
Cumulative epinephrine dose	424 (13.4)	0 (0.0)	0 (0.0)	0 (0.0)	N/A
Time to epinephrine	1,128 (35.6)	890 (36.0)	32 (43.2)	858 (35.8)	0.188
Initial rhythm	0 (0.0)	0 (0.0)	0 (0.0)	0 (0.0)	N/A
Arrest location	0 (0.0)	0 (0.0)	0 (0.0)	0 (0.0)	N/A
Sustained ROSC	0 (0.0)	0 (0.0)	0 (0.0)	0 (0.0)	N/A
Early mortality within 24 h	0 (0.0)	0 (0.0)	0 (0.0)	0 (0.0)	N/A
Medication history/exposure classification	356 (11.2)	0 (0.0)	0 (0.0)	0 (0.0)	N/A

Missingness counts in the screened cohort are variable-level counts and are not mutually exclusive. Figure 1 reports the primary exclusion reason assigned to each excluded case.

### 2.6 Data collection and variables

Demographic variables included age and sex. Comorbid conditions recorded were hypertension, type 2 diabetes mellitus, coronary artery disease, malignancy, and chronic beta-blocker therapy. Comorbidities were extracted from coded diagnoses and problem lists in the electronic medical record, supplemented by outpatient referral letters and medication lists (e.g., antianginal or antiplatelet therapy as a proxy indicator for coronary artery disease) when coded diagnoses were absent; this retrospective, chart-based extraction approach is a recognized source of potential comorbidity misclassification and is discussed as a limitation below. Arrest-related variables included arrest location (OHCA vs. IHCA), initial cardiac rhythm classified as shockable (ventricular fibrillation or pulseless ventricular tachycardia) or nonshockable (asystole or pulseless electrical activity), and timing of epinephrine administration relative to CPR initiation. Total CPR duration and cumulative epinephrine dose were fully documented within the final analytic cohort (0% missing in the 2,472 included patients); missingness in these variables was largely confined to cases that were excluded from the analytic cohort for other reasons (Table 2, Supplementary Missingness Table), and did not differ significantly between exposure groups for the remaining partially documented variable, time to epinephrine administration (43.2% missing among alpha-blocker users vs. 35.8% missing among non-users, *P* = 0.188).

**Table 2:** Descriptive resuscitation outcomes by individual alpha-1 adrenergic blocker agent.

Agent	n	Refractory by definition, *n* (%)	ROSC, *n* (%)	Early mortality, *n* (%)
Doxazosin	27	15 (55.6)	11 (40.7)	19 (70.4)
Alfuzosin	21	10 (47.6)	10 (47.6)	16 (76.2)
Silodosin	19	14 (73.7)	5 (26.3)	14 (73.7)
Unknown/unspecified	7	6 (85.7)	1 (14.3)	5 (71.4)

Numbers per agent are insufficient for formal statistical subgroup comparison.

### 2.7 Outcome measures

Two related but distinct resuscitation-failure outcomes were assessed to address concerns regarding conflation of endpoints. The primary outcome was an operationally defined refractory cardiac arrest indicator, generated independently in the dataset (variable refractory_by_definition) as a cumulative epinephrine dose of ≥3 mg and/or a CPR duration of ≥20 min occurring during the resuscitation course, regardless of eventual outcome; this operational definition is not the arithmetic inverse of ROSC status, because a patient could meet the prolonged-resuscitation/high-dose criteria and still ultimately achieve ROSC after further intervention, or could achieve early ROSC without ever meeting the prolonged-therapy threshold. A secondary, closely related outcome, failure to achieve sustained ROSC, was defined as the absence of restoration of spontaneous circulation with palpable pulses and measurable blood pressure by the end of the resuscitation attempt; this outcome is, by construction, the complement of ROSC and is reported separately from the operational refractory-arrest indicator to allow direct comparison of the two constructs. Early mortality, defined as death in the emergency department or within the first 24 h following resuscitation, was assessed as an additional secondary outcome.

### 2.8 Statistical analysis

Continuous variables were summarized as means with standard deviations or medians with interquartile ranges, as appropriate, and were compared using Welch t-tests or Mann–Whitney U tests depending on distribution. Categorical variables were expressed as counts and percentages and were compared using chi-square or Fisher's exact tests when expected cell counts were small. Standardized mean differences (SMD) were calculated for all baseline variables in addition to *P* values, given the highly unequal group sizes (*n* = 74 vs. *n* = 2,398); an SMD greater than 0.10 was considered indicative of meaningful imbalance.

Associations between chronic alpha-1 adrenergic blocker use and clinical outcomes were evaluated using unadjusted odds ratios (OR) with 95% confidence intervals (CI), absolute risk differences, and risk ratios. Given the limited number of exposed patients and incomplete availability of several resuscitation-level covariates, comprehensive multivariable or propensity score-based adjustment was not considered statistically reliable. Instead, a single prespecified parsimonious multivariable logistic regression model, adjusting only for age, sex, arrest location (OHCA vs. IHCA), and initial cardiac rhythm (shockable vs. non-shockable), was applied uniformly to all four outcomes (operationally defined refractory arrest, failure to achieve sustained ROSC, ROSC, and early mortality) to minimize overfitting while assessing robustness of the unadjusted findings. Adjusted odds ratios, 95% confidence intervals, and *P* values from this model are reported in Table 3. A two-sided *P* value <0.05 was considered statistically significant. Analyses were conducted using IBM SPSS Statistics (version 26.0) and cross-validated using Python (statsmodels, version 0.14). In addition, a sensitivity analysis was conducted in the subset of patients with complete data on CPR duration and cumulative epinephrine dose, which corresponded to the full final analytic cohort given the complete documentation of these variables in the included patients.

**Table 3:** Association between chronic alpha-1 adrenergic blocker exposure and early resuscitation outcomes, including absolute risk differences, risk ratios, and adjusted odds ratios.

Outcome	Alpha-blocker (+) (*n* = 74)	Alpha-blocker (–) (*n* = 2,398)	Absolute risk difference (95% CI)	Risk ratio (95% CI)	Unadjusted OR (95% CI)	Adjusted OR (95% CI)	Adjusted *P* value
Refractory cardiac arrest (operational definition)	45 (60.8)	840 (35.0)	25.8% (14.5 to 37.1)	1.74 (1.43–2.10)	2.88 (1.79–4.62)	3.06 (1.88–4.97)	<0.001
Failure to achieve sustained ROSC	47 (63.5)	1,002 (41.8)	21.7% (10.6 to 32.9)	1.52 (1.27–1.82)	2.43 (1.50–3.92)	2.64 (1.62–4.31)	<0.001
Return of spontaneous circulation (ROSC)	27 (36.5)	1,396 (58.2)	−21.7% (−32.9 to −10.6)	0.63 (0.46–0.85)	0.41 (0.26–0.67)	0.38 (0.23–0.62)	<0.001
Early mortality within 24 h	54 (73.0)	636 (26.5)	46.5% (36.2 to 56.7)	2.75 (2.36–3.21)	7.48 (4.44–12.59)	7.15 (4.20–12.15)	<0.001

Adjusted ORs were derived from a parsimonious logistic regression model including alpha-blocker exposure, age, sex, arrest location, and initial cardiac rhythm. OR, odds ratio; CI, confidence interval; ROSC, return of spontaneous circulation.

## 3 Results

A total of 3,168 cardiac arrest cases were screened during the study period. Of these, 696 cases were excluded according to the disaggregated reasons summarized above, leaving 2,472 patients for the final analysis (Figure 1). Among the included patients, 74 (3.0%) were receiving chronic alpha-1 adrenergic blocker therapy at the time of cardiac arrest, and all 74 of these patients, together with all 2,398 non-users, received intravenous epinephrine during CPR, directly addressing the question of epinephrine exposure in the alpha-1 blocker group.

### 3.1 Baseline characteristics

Baseline characteristics of the study population, now including SMD values alongside *P* values in a dedicated column as requested, are summarized in Table 4. Patients in the alpha-blocker group were younger on average (66.3 ± 9.6 vs. 77.5 ± 11.0 years; SMD 1.087) and more frequently male (70.3% vs. 51.1%; SMD 0.401). Hypertension was more common among alpha-blocker users (68.9% vs. 32.8%; SMD 0.775), whereas coronary artery disease (5.4% vs. 65.6%; SMD 1.619) and type 2 diabetes mellitus (10.8% vs. 42.3%; SMD 0.763) were markedly more prevalent in the non-alpha-blocker group. Malignancy was somewhat more common among alpha-blocker users (13.5% vs. 7.0%; SMD 0.217). Shockable initial rhythms were observed more frequently among alpha-blocker users (16.2% vs. 5.7%; SMD 0.343). Arrest location (OHCA vs. IHCA) and beta-blocker use did not differ meaningfully between groups (SMD <0.10 for both). These SMD values confirm that several comorbidities, most notably coronary artery disease, show large imbalances that exceed conventional thresholds for confounding concern; this pattern is addressed explicitly in the Discussion and Limitations, where retrospective coding artifacts and incomplete comorbidity capture are considered as plausible contributors alongside genuine clinical differences between exposure groups.

**Table 4:** Baseline characteristics of the study population according to chronic alpha-1 adrenergic blocker use, with standardized mean differences.

Variable	Alpha-blocker (+) (*n* = 74)	Alpha-blocker (–) (*n* = 2,398)	*P* value	SMD
Age, mean ± SD	66.3 ± 9.6	77.5 ± 11.0	<0.001	1.087
Male sex, *n* (%)	52 (70.3)	1,225 (51.1)	0.001	0.401
Hypertension, *n* (%)	51 (68.9)	786 (32.8)	<0.001	0.775
Coronary artery disease, *n* (%)	4 (5.4)	1,574 (65.6)	<0.001	1.619
Type 2 diabetes mellitus, *n* (%)	8 (10.8)	1,014 (42.3)	<0.001	0.763
Malignancy, *n* (%)	10 (13.5)	167 (7.0)	0.031	0.217
Beta-blocker use, *n* (%)	38 (51.4)	1,329 (55.4)	0.488	0.082
OHCA, *n* (%)	40 (54.1)	1,266 (52.8)	0.831	0.025
IHCA, *n* (%)	34 (45.9)	1,132 (47.2)	0.831	0.025
Shockable rhythm, *n* (%)	12 (16.2)	136 (5.7)	0.001	0.343

OHCA, out-of-hospital cardiac arrest; IHCA, in-hospital cardiac arrest; SD, standard deviation; SMD, standardized mean difference (values >0.10 indicate meaningful imbalance).

### 3.2 Primary outcome

Using the operational refractory-arrest indicator captured independently in the dataset, refractory cardiac arrest occurred in 45 of 74 patients (60.8%) in the alpha-blocker group and in 840 of 2,398 patients (35.0%) in the non-alpha-blocker group (unadjusted OR 2.88, 95% CI 1.79–4.62; adjusted OR 3.06, 95% CI 1.88–4.97, *P* < 0.001). For comparison, failure to achieve sustained ROSC, a related but conceptually distinct endpoint, occurred in 47 of 74 patients (63.5%) versus 1,002 of 2,398 patients (41.8%) (unadjusted OR 2.43, 95% CI 1.50–3.92; adjusted OR 2.64, 95% CI 1.62–4.31, *P* < 0.001). The absolute risk difference for the operational refractory-arrest indicator was 25.8 percentage points (95% CI 14.5 to 37.1), with a risk ratio of 1.74 (95% CI 1.43–2.10) (Table 3).

### 3.3 Secondary outcomes

Return of spontaneous circulation (ROSC) was achieved in 27 patients (36.5%) receiving alpha-blockers and in 1,396 patients (58.2%) not receiving alpha-blockers. Chronic alpha-blocker use was associated with lower odds of ROSC (unadjusted OR 0.41, 95% CI 0.26–0.67; adjusted OR 0.38, 95% CI 0.23–0.62, *P* < 0.001), corresponding to an absolute risk difference of −21.7 percentage points (95% CI −32.9 to −10.6) and a risk ratio of 0.63 (95% CI 0.46–0.85).

Early mortality, defined as death in the emergency department or within the first 24 h following resuscitation, occurred in 54 patients (73.0%) in the alpha-blocker group and in 636 patients (26.5%) in the non-alpha-blocker group. The unadjusted odds of early mortality were higher among alpha-blocker users (OR 7.48, 95% CI 4.44–12.59; adjusted OR 7.15, 95% CI 4.20–12.15, *P* < 0.001), with an absolute risk difference of 46.5 percentage points (95% CI 36.2 to 56.7) and a risk ratio of 2.75 (95% CI 2.36–3.21) (Table 3).

### 3.4 Adjusted and sensitivity analyses

**Figure 2:**
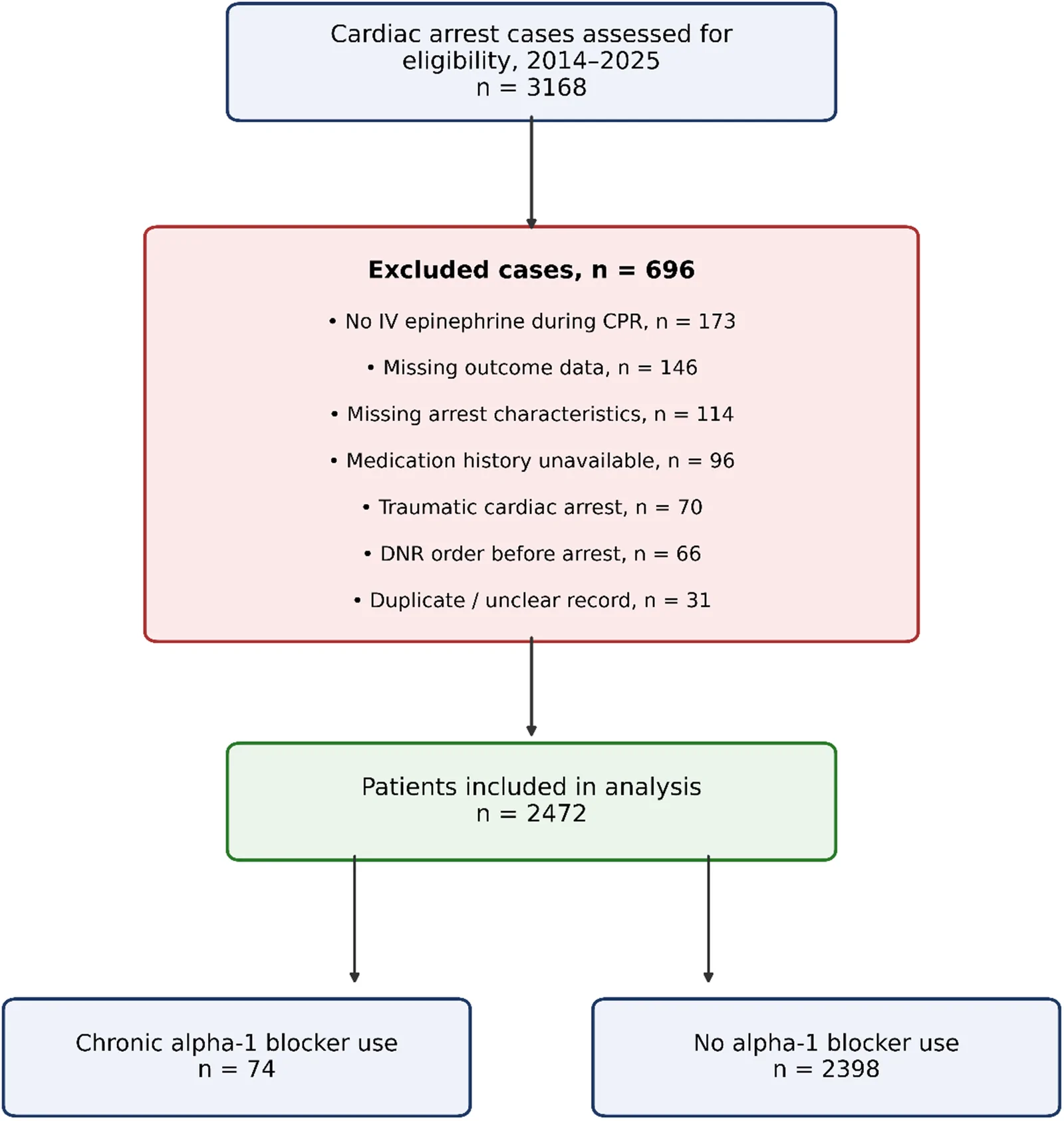
Forest plot of adjusted odds ratios (with 95% confidence intervals) for chronic alpha-1 adrenergic blocker exposure across four resuscitation outcomes, adjusted for age, sex, arrest location, and initial cardiac rhythm.

In the prespecified parsimonious adjusted model, chronic alpha-1 adrenergic blocker use remained associated with higher odds of the operationally defined refractory arrest indicator (adjusted OR 3.06, 95% CI 1.88–4.97), higher odds of failure to achieve sustained ROSC (adjusted OR 2.64, 95% CI 1.62–4.31), lower odds of ROSC (adjusted OR 0.38, 95% CI 0.23–0.62), and higher odds of early mortality (adjusted OR 7.15, 95% CI 4.20–12.15), after adjustment for age, sex, arrest location, and initial cardiac rhythm (all *P* < 0.001). These four adjusted estimates are displayed graphically in Figure 2. The direction and magnitude of these associations were consistent with, and numerically slightly larger than, the corresponding unadjusted estimates, indicating that the observed associations were not attenuated by adjustment for these core covariates; nevertheless, given the substantial baseline imbalance documented by the SMD values in Table 4, residual confounding from unmeasured or incompletely captured variables, particularly coronary artery disease burden, cannot be excluded.

Because CPR duration and cumulative epinephrine dose were fully documented in the final analytic cohort (0% missing among the 2,472 included patients), the previously described sensitivity analysis restricted to patients with complete resuscitation-level data corresponds to the entire final cohort rather than a smaller subset. Within this cohort, both CPR duration and cumulative epinephrine dose were numerically higher in the alpha-blocker group, consistent with the higher observed rate of the operationally defined refractory resuscitation profile in this group.

### 3.5 Missing data

Missingness for key resuscitation variables is summarized in Table 1. Among the 3,168 screened cases, CPR duration and cumulative epinephrine dose were missing in 12.8% and 13.4% of cases, respectively; however, these missing values occurred exclusively among the 696 cases subsequently excluded from the analytic cohort, and both variables were completely documented (0% missing) among the 2,472 patients retained for analysis. Medication history required for exposure classification was missing in 11.2% of screened cases but, by design, was complete in the final cohort, since incomplete medication history was itself an exclusion criterion. The only resuscitation variable with residual missingness in the final cohort was time to epinephrine administration, missing in 890 of 2,472 patients (36.0%); this missingness did not differ significantly between alpha-blocker users (43.2%) and non-users (35.8%) (*P* = 0.188), suggesting that differential missingness by exposure status is an unlikely source of bias for this variable.

### 3.6 Agent-specific descriptive outcomes

Doxazosin, alfuzosin, and silodosin differ in pharmacokinetic profile and alpha-1 receptor subtype selectivity. A descriptive breakdown by individual agent is presented in Table 2; however, with only 27, 21, and 19 patients receiving doxazosin, alfuzosin, and silodosin, respectively (and 7 with an unspecified agent), these numbers are too small to support a statistically reliable agent-specific subgroup analysis, and the reported outcome frequencies should be interpreted purely as descriptive.

## 4 Discussion

In this retrospective exploratory cohort study, chronic alpha-1 adrenergic blocker therapy was associated with altered early resuscitation profiles among adult cardiac arrest patients treated with epinephrine. Specifically, alpha-blocker exposure was associated with higher rates of an operationally defined refractory resuscitation pattern, lower rates of ROSC, and increased early mortality, and these associations persisted, with similar or slightly greater magnitude, after adjustment for age, sex, arrest location, and initial cardiac rhythm. These findings were observed in a large and heterogeneous cardiac arrest population managed under standardized resuscitation protocols; however, they should be interpreted cautiously as observational and hypothesis-generating signals rather than evidence of a causal pharmacologic effect, particularly given the marked baseline imbalance documented by SMD analysis and the small number of exposed patients.

From a mechanistic perspective, the observed associations are biologically plausible with respect to the alpha-1-mediated component of epinephrine action, because vasoconstriction and the resulting increases in aortic diastolic pressure and coronary perfusion pressure during CPR are thought to represent key determinants of successful resuscitation and ROSC (1, 2). However, epinephrine's resuscitative efficacy is not attributable to alpha-1 stimulation alone. Beta-adrenergic stimulation, and beta-2 adrenoceptor activation specifically, has been proposed to contribute importantly to pulse generation in the sinoatrial node and to restoration of contractile function in ischemic cardiomyocytes, a mechanism that may help explain why epinephrine, rather than a pure alpha-1 agonist such as phenylephrine, remains the vasopressor recommended in advanced cardiac life support guidelines (3). At the same time, beta-adrenergic stimulation may contribute to potentially unfavorable physiological effects, including increased myocardial oxygen consumption, post-resuscitation myocardial dysfunction, and arrhythmogenesis (4–6). Chronic alpha-1 blockade would not be expected to attenuate these beta-adrenergic actions of epinephrine; therefore, if chronic alpha-1 blockade meaningfully alters resuscitation profiles, the most plausible pharmacodynamic pathway is selective attenuation of the alpha-1-mediated vasoconstrictive component of epinephrine's action, leaving beta-adrenergic effects comparatively intact. Under the profoundly hypoperfused conditions of cardiac arrest, even partial attenuation of alpha-adrenergic vascular responsiveness could theoretically influence vasopressor effectiveness during resuscitation. Nevertheless, direct clinical evidence demonstrating that chronic outpatient alpha-blockade meaningfully alters responsiveness to standard-dose epinephrine during CPR remains limited, and the present study did not include real-time hemodynamic measurements (e.g., arterial systolic, diastolic, or mean pressures, or aortic pressure tracings) that would be needed to directly characterize this mechanism. Accordingly, the present findings should be viewed as mechanistically suggestive rather than confirmatory, and future prospective studies incorporating invasive or non-invasive hemodynamic monitoring during CPR are needed to test this hypothesis directly.

An interesting observation in the present study was that the alpha-blocker group did not appear uniformly higher risk across several baseline descriptors. Patients receiving alpha-blockers were younger and demonstrated a higher proportion of shockable initial rhythms, both features generally associated with more favorable cardiac arrest outcomes. Despite this, early resuscitation profiles remained less favorable in the exposed group. Although this pattern does not establish a causal pharmacodynamic interaction, it suggests that baseline clinical risk alone may not fully explain the observed associations. At the same time, the standardized mean differences reported in Table 4 confirm a markedly lower prevalence of coronary artery disease and diabetes mellitus among alpha-blocker users (SMD 1.619 and 0.763, respectively), well above conventional thresholds for meaningful imbalance. Given that alpha-1 adrenergic blockers are frequently prescribed to older patients with hypertension who might otherwise be expected to carry substantial atherosclerotic burden, this pattern raises specific concern for retrospective comorbidity coding artifacts or incomplete capture of diagnoses in the electronic record, rather than necessarily reflecting a true underlying difference in cardiovascular risk. Comorbidities in this study were extracted primarily from coded diagnoses and problem lists, supplemented by medication-based proxies when coded diagnoses were unavailable; while this approach is consistent with common retrospective methodology, it is inherently susceptible to underascertainment, particularly for conditions such as coronary artery disease that may be documented inconsistently outside of dedicated cardiology encounters. These baseline imbalances, together with the residual possibility of unmeasured confounding related to prescribing patterns and underlying cardiovascular risk profiles, further emphasize the exploratory nature of the present findings and argue against any causal interpretation of the observed associations.

The concept of refractory or vasopressor-resistant cardiac arrest has received increasing attention in recent years, particularly in the setting of prolonged resuscitation and extracorporeal cardiopulmonary resuscitation strategies (10–12). In the present study, the operationally defined refractory-arrest indicator and failure to achieve sustained ROSC were assessed as related but distinct constructs, in response to reviewer concern that these outcomes might otherwise be conflated. As shown in Table 3, the two endpoints yielded numerically similar but non-identical estimates (adjusted OR 3.06 for the operational refractory-arrest indicator versus 2.64 for ROSC failure), consistent with partial but incomplete overlap between the two definitions; this pattern supports treating the operationally defined refractory-arrest indicator as a genuine, prospectively captured dataset variable rather than a construct that is mathematically equivalent to ROSC failure. Most previous investigations in this area have focused on resuscitation timing, escalation strategies, or mechanical circulatory support, while relatively limited attention has been directed toward chronic outpatient pharmacologic factors that may influence vasopressor responsiveness during CPR. Although several observational studies have evaluated the relationship between pre-arrest beta-blocker therapy and cardiac arrest outcomes, reported findings remain inconsistent, and data regarding chronic alpha-1 adrenergic blocker exposure remain sparse (11–13). To our knowledge, few clinical studies have specifically explored the possible relationship between chronic alpha-adrenergic blockade and epinephrine responsiveness during cardiac arrest, highlighting an underexplored area within resuscitation pharmacology.

Importantly, the present findings should primarily be interpreted as signal-generating rather than practice changing. Cardiac arrest outcomes are influenced by numerous patient-, treatment-, and system-level variables, many of which cannot be fully captured within retrospective datasets. Consequently, the observed associations may reflect a complex interaction between baseline patient characteristics, arrest physiology, and pharmacologic responsiveness rather than an isolated medication effect alone. The findings therefore do not support modifications to current resuscitation guidelines or vasopressor dosing strategies, and in particular do not support substitution of alternative vasopressor agents in patients on chronic alpha-1 blocker therapy. One potential implication of these observations is whether non-adrenergic vasopressor strategies, such as vasopressin-based approaches, might behave differently in selected patient populations with chronic adrenergic modulation. However, the present study was not designed to evaluate therapeutic implications or alternative vasopressor selection, and no inference regarding treatment modification can be drawn from the current dataset.

Given the limited number of exposed patients and incomplete availability of certain resuscitation-level covariates (specifically, time to epinephrine administration), only a single prespecified parsimonious adjusted analysis, incorporating age, sex, arrest location, and initial rhythm, was performed. More comprehensive multivariable adjustment, particularly incorporating markers of cardiovascular comorbidity burden, or propensity score-based approaches, were not considered sufficiently reliable within the current data structure given the extreme imbalance in group sizes (*n* = 74 vs. *n* = 2,398) and the risk of model overfitting or non-convergence. Despite these limitations, the study highlights a clinically plausible and relatively underexplored area within resuscitation pharmacology. Further prospective multicenter investigations incorporating detailed physiologic monitoring, pharmacologic exposure characterization (including confirmed dosing, adherence, and time since last dose), and advanced adjusted analyses conducted in collaboration with biostatisticians are needed to better clarify whether chronic adrenergic modulation meaningfully influences vasopressor responsiveness during cardiac arrest.

## 5 Limitations

Several limitations should be considered when interpreting the present findings. First, because of the retrospective observational design, the observed associations cannot be interpreted as causal and should instead be viewed as exploratory and hypothesis-generating. In addition, although the overall cohort size was relatively large, the number of patients receiving chronic alpha-1 adrenergic blocker therapy remained limited (*n* = 74 versus *n* = 2,398), which reduces statistical precision, increases susceptibility to the influence of a small number of individual cases, and, as illustrated by the standardized mean differences in Table 4, is associated with substantial baseline imbalance between exposure groups that cannot be fully addressed through a parsimonious four-covariate adjustment model alone. The revised statistical analyses were additionally reviewed for consistency, and the final model was intentionally restricted to a prespecified parsimonious adjustment strategy because of the limited number of exposed patients.

Cardiac arrest outcomes are influenced by numerous patient-, treatment-, and system-level variables. Although standardized institutional resuscitation protocols were used throughout the study period, several potentially important resuscitation-quality parameters, including no-flow and low-flow times, witnessed status, bystander CPR, real-time hemodynamic measurements (e.g., arterial blood pressure, aortic systolic/diastolic pressure, or mean arterial pressure during CPR), and detailed post-resuscitation care variables, were either unavailable or incompletely documented within the retrospective dataset and could not be incorporated into the present analysis. The absence of real-time hemodynamic data in particular limits the ability to directly demonstrate the proposed pharmacodynamic mechanism linking alpha-1 blockade to attenuated vasopressor responsiveness, and this should be regarded as a central limitation of the mechanistic interpretation offered in the Discussion.

Medication adherence and the exact timing of the final alpha-blocker dose before cardiac arrest could not be verified reliably, and exposure was defined using an active outpatient prescription as a pragmatic proxy for chronic use rather than a biochemically confirmed measure of pharmacological exposure at the time of arrest. Potential pharmacodynamic heterogeneity among individual alpha-1 adrenergic antagonists was explored descriptively (Table 2) but could not be evaluated with formal subgroup analysis because of small per-agent sample sizes. Furthermore, residual confounding remains likely, including confounding related to prescribing patterns and underlying cardiovascular risk profiles; the markedly lower prevalence of coronary artery disease and diabetes mellitus among alpha-blocker users, despite these patients being older on average than would be expected for a lower-comorbidity subgroup relative to national prescribing patterns for alpha-1 blockers, raises the possibility of retrospective comorbidity coding artifacts, incomplete diagnostic capture, or genuine selection effects related to prescribing practice; the present data cannot fully distinguish between these explanations. Although a prespecified parsimonious adjusted analysis was performed, more comprehensive multivariable or propensity-based approaches were not considered sufficiently reliable within the available data structure given the extreme group-size imbalance.

Finally, this was a single-center study conducted within a specific healthcare environment, which may limit external validity. Although CPR duration and cumulative epinephrine dose were fully documented within the final analytic cohort, other resuscitation-quality covariates, most notably time to epinephrine administration, remained incompletely documented, and this residual missingness, together with the small exposed group size, constrains the granularity of subgroup and sensitivity analyses that could be performed. Nevertheless, the present study reflects a large real-world cardiac arrest cohort and explores a clinically plausible but relatively underinvestigated aspect of resuscitation pharmacology. The findings should therefore be interpreted primarily as signal-generating observations that may help inform future prospective multicenter investigations incorporating richer physiologic and pharmacologic data, ideally designed and analyzed in direct collaboration with biostatisticians from the outset.

## 6 Conclusions

In this retrospective exploratory cohort study, chronic alpha-1 adrenergic blocker therapy was associated with altered early resuscitation profiles among cardiac arrest patients treated with epinephrine, an association that persisted after adjustment for age, sex, arrest location, and initial cardiac rhythm. These findings should be interpreted cautiously in light of the small number of exposed patients, the marked baseline imbalance in cardiovascular comorbidity burden between exposure groups, and the inherent limitations of retrospective comorbidity ascertainment, and should not be viewed as evidence that routine outpatient alpha-adrenergic blockade definitively attenuates the clinical effects of epinephrine during cardiopulmonary resuscitation. Rather, the observed associations raise the possibility that chronic adrenergic modulation, superimposed on epinephrine's combined alpha- and beta-adrenergic mechanisms of action, may represent a clinically relevant but underexplored pharmacodynamic factor during cardiac arrest resuscitation. Further prospective multicenter investigations incorporating richer physiologic data, confirmed pharmacologic exposure, and advanced adjusted analyses conducted with formal biostatistical collaboration are needed to better clarify this relationship.

## Data Availability

The raw data supporting the conclusions of this article will be made available by the authors, without undue reservation.
